# Maturation-Dependent Differences in the Re-innervation of the Denervated Dentate Gyrus by Sprouting Associational and Commissural Mossy Cell Axons in Organotypic Tissue Cultures of Entorhinal Cortex and Hippocampus

**DOI:** 10.3389/fnana.2021.682383

**Published:** 2021-05-28

**Authors:** Mandy H. Paul, Lars Hildebrandt-Einfeldt, Viktor J. Beeg Moreno, Domenico Del Turco, Thomas Deller

**Affiliations:** Institute of Clinical Neuroanatomy, Dr. Senckenberg Anatomy, Neuroscience Center, Goethe-University Frankfurt, Frankfurt, Germany

**Keywords:** calretinin, perforant path transection, organotypic culture, dentate gyrus, layer-specificity

## Abstract

Sprouting of surviving axons is one of the major reorganization mechanisms of the injured brain contributing to a partial restoration of function. Of note, sprouting is maturation as well as age-dependent and strong in juvenile brains, moderate in adult and weak in aged brains. We have established a model system of complex organotypic tissue cultures to study sprouting in the dentate gyrus following entorhinal denervation. Entorhinal denervation performed after 2 weeks postnatally resulted in a robust, rapid, and very extensive sprouting response of commissural/associational fibers, which could be visualized using calretinin as an axonal marker. In the present study, we analyzed the effect of maturation on this form of sprouting and compared cultures denervated at 2 weeks postnatally with cultures denervated at 4 weeks postnatally. Calretinin immunofluorescence labeling as well as time-lapse imaging of virally-labeled (AAV2-hSyn1-GFP) commissural axons was employed to study the sprouting response in aged cultures. Compared to the young cultures commissural/associational sprouting was attenuated and showed a pattern similar to the one following entorhinal denervation in adult animals *in vivo*. We conclude that a maturation-dependent attenuation of sprouting occurs also *in vitro*, which now offers the chance to study, understand and influence maturation-dependent differences in brain repair in these culture preparations.

## Introduction

The reorganization of denervated brain regions is seen in many pathological conditions in which some form of structural damage has occurred (Steward, 1991; Deller and Frotscher, 1997; Perederiy and Westbrook, 2013). Following a local lesion, connected brain regions are denervated and become sites of secondary brain injury. Thus, a local lesion of the brain is not really “local” but rather the spot where the neuronal network has been disrupted. Neurons respond to this challenge to the network by rewiring their connections aiming at re-establishing homeostasis and information throughput (Steward, 1994; Vlachos et al., 2012a,b, 2013a; Willems et al., 2016; Yap et al., 2020). This may eventually result in a partial restoration of function, which may even be strengthened by training or specific rehabilitation techniques (Fawcett, 2009; Garcia-Alias et al., 2009; Maier et al., 2009; Martin, 2012; Filli and Schwab, 2015).

Denervation–induced reorganization of the brain involves different cellular players as well as a number of not yet fully understood mechanisms (Steward, 1991; Deller and Frotscher, 1997; Perederiy and Westbrook, 2013). A major reorganization phenomenon that has been studied for many years is collateral sprouting (Steward, 1991; Deller et al., 1995, 1996a). Although we do not yet fully understand all of the rules of this mechanism, sprouting axons are able to replace some of the synapses that were lost thus enabling denervated neurons to continue functioning. Beneficial effects of sprouting include restoring a denervated neuron to its physiological firing range, maintaining the dendritic arbor of a denervated neuron, and, in the case of sprouting axons that are homologous to those that were lost, restoration of information flow (Steward, 1991; Deller and Frotscher, 1997).

Collateral sprouting is, however, not the same at all ages. It has been shown previously that maturation plays an important role: juvenile animals (2–3 weeks old) show a much faster and much more extensive sprouting response than adults (Gall and Lynch, 1980, 1981; Gall et al., 1980; McWilliams and Lynch, 1983). Moreover, a recent article showed that sprouting diminishes even further with aging (Askvig and Watt, 2019). Several years ago, we established an *in vitro* sprouting model using organotypic slice cultures of the entorhinal cortex and hippocampus (Prang et al., 2003; Del Turco and Deller, 2007; Del Turco et al., 2019). In these complex cultures, in which the dentate gyrus is usually de-entorhinated during the first weeks postnatally, a fast and extensive sprouting response similar to the one reported for juvenile rats (Gall and Lynch, 1980, 1981; Gall et al., 1980; McWilliams and Lynch, 1983) is observed (Prang et al., 2003; Del Turco and Deller, 2007; Del Turco et al., 2019). Since organotypic slice cultures continue to mature also *in vitro* (Li et al., 1995; Dailey and Smith, 1996; Ziv and Smith, 1996), we wondered whether the strong sprouting response we see in young culture preparations is attenuated in older cultures. To cover approximately the same time frame * in vitro* as the one studied previously *in vivo* (e.g., Gall and Lynch, 1980), we compared organotypic entorhino-hippocampal (EC-H) slice cultures lesioned at 14 days *in vitro* with EC-H slice cultures lesioned at 28 days *in vitro* and investigated the extent of the commissural/associational sprouting response 2 weeks later. Indeed, in older slice cultures commissural/associational sprouting was much weaker, suggesting that a maturation-dependent attenuation of collateral sprouting is also seen *in vitro* where it can now be further investigated.

## Materials and Methods

### Animals

The generation of organotypic entorhino-hippocampal tissue cultures from C57Bl6/J mice was performed in accordance with the German animal welfare law and had been declared to the Animal Welfare Officer of Goethe-University, Faculty of Medicine (Wa-2014-35). Mice were bred and housed at the animal facility of the Goethe-University Hospital Frankfurt, and were maintained on a 12 h light/dark cycle with food and water available *ad libitum*. Every effort was made to minimize the distress and pain of animals.

### Organotypic Tissue Cultures

Entorhino-hippocampal tissue cultures were prepared from mouse brains of C57Bl6/J mice at postnatal days 3–5 according to previously published protocols (Prang et al., 2003; Del Turco and Deller, 2007; Del Turco et al., 2019). For brain dissection, an ice-cold preparation medium [Minimal essential medium (MEM, Gibco) containing 2 mM Glutamax (Gibco), pH 7.3] was used. Slices (300–350 μm) were cut using a Leica vibratome (VT1200S, Leica). Organotypic tissue cultures were maintained on porous-membrane filter inserts (Millicell-CM, Millipore) and incubated in a humidified atmosphere with 5% CO_2_ at 35°C. Medium for cultivation contained 42% MEM, 25% basal medium eagle (Gibco), 25% heat-inactivated normal horse serum (Gibco), 2.5% HEPES buffer solution (Invitrogen), 0.15% bicarbonate (Invitrogen), 0.675% glucose (Sigma–Aldrich), 0.1 mg/ml streptomycin (Sigma–Aldrich), 100 U/ml penicillin (Sigma–Aldrich) and 2 mM Glutamax. The pH was adjusted to 7.3 and the medium was replaced every 2–3 days. Organotypic tissue cultures were incubated for up to 42 days *in vitro* (DIV).

### Perforant Path Lesion *In vitro*

Cultures were allowed to mature for 14 or 28 DIV, respectively. Using a sterile scalpel blade the entorhinal cortex was cut away from the culture and removed from the culture dish, as described (Del Turco et al., 2019). After the lesion, cultures were placed back into the incubator and maintained for 14 days post-lesion.

### Adeno-Associated Virus Production

Pseudotyped adeno-associated viral (AAV) particles were generated using a helper virus-free packaging method as previously described with minor modifications (Hildebrandt-Einfeldt et al., 2021). HEK293T cells were simultaneously transfected with pDP1rs (Plasmid Factory), pDG (Plasmid Factory), and either an AAV2-hSyn1-GFP or AAV2-hSyn1-tdTomato vector plasmid (Shevtsova et al., 2005; 12:8:5) using calcium phosphate precipitation (protocol adapted from Grimm et al., 1998; Grimm, 2002). Cells were collected 48 h after transfection, washed twice with Phosphate Buffered Saline (PBS), centrifuged at 8,00–1,200 rpm for 5–10 min and re-suspended in PBS. Recombinant viral particles within the cells were released by four freeze-and thaw cycles and the supernatant was centrifuged at 10,000 rpm for 10 min to remove cell debris. The final supernatant was collected, aliquoted, and stored at −80°C.

### Viral Labeling

To transduce and thus label neurons in the EC, slice cultures were injected (DIV 3–5) with AAV2-hSyn1-GFP or AAV2-hSyn1-tdTomato virus. The EC was visually identified in slices based on its location and morphology and injections were performed under visual control, as previously described (Hildebrandt-Einfeldt et al., 2021).

To label hilar mossy cells, tissue cultures were transduced with an adeno-associated virus serotype 2 (AAV2) containing GFP under the human Synapsin 1 promoter (AAV2-hSyn1-GFP). Injections were performed using an injection pipette pulled from thin-walled borosilicate capillaries (Harvard Apparatus, 30-0066). Pipettes were held by a head stage with a HL-U holder (Axon Instruments) and positioned using a micro-manipulator (Luigs and Neumann). Approximately 0.05–0.1 μl of AAV2-hSyn1-GFP was injected directly into the hilar region of the dentate gyrus using a syringe. Tissue cultures were visualized with an upright microscope (Nikon Y-TV55) using a 10× water immersion objective lens (Nikon Plan Fluor, NA 0.30). All injections were performed 3–4 days after the tissue cultures were prepared.

### Time-Lapse Imaging of Organotypic Tissue Cultures

Live imaging of tissue cultures was performed as previously described (Del Turco et al., 2019). The membrane insert with the cultures was placed into a 30 mm petri dish that contained imaging medium (37°C) which consisted of NaCl 129 mM, KCl 4 mM, MgCl_2_ 1 mM, CaCl_2_ 2 mM, glucose 4.2 mM, HEPES 10 mM, Trolox 0.1 mM, streptomycin 0.1 mg/ml, penicillin 100 U/ml; pH 7.4. The osmolarity of the imaging medium was adjusted with sucrose to the osmolarity of the incubation medium. Imaging was done with an upright confocal microscope (Olympus Fluoview VF3000; 488 nm excitation laser) equipped with a temperature-regulated stage (37°C), using a 20× water immersion objective lens (0.3 NA; Olympus US2) to visualize tissue cultures and to identify AAV-labeled mossy cells in the hilus of the DG. Image stacks (approximately 20 images per stack; z-axis interval between consecutive frames: 5 mm) of DG regions were obtained at a resolution of 1,024 × 1,024 pixels. Tissue cultures were imaged on 28 and 42 days *in vitro* for less than 10 min per culture in order to keep exposure time and phototoxic damage minimal. GFP-intensity levels of AAV-injected cultures were equalized at DIV 42 using Adobe Photoshop CS6 (Version 13.0.1 x 64) to account for differences in GFP expression between cultures.

### Immunofluorescence

Immunofluorescence was performed as described (Del Turco et al., 2019; Hildebrandt-Einfeldt et al., 2021) with minor modifications. For cutting on a vibratome (VT 1000S, Leica), cultures were fixed in 4% paraformaldehyde (PFA) in 0.1 M PBS (pH 7.4) for 4 h. After several washes in buffer they were re-sliced into 30 μm sections. For cutting on a cryostat (CM3050 S, Leica), cultures were fixed in 4% PFA in 0.1 M PBS (pH 7.4) and 4% sucrose for 1 h at room temperature (RT), followed by 2% PFA and 30% sucrose in PBS overnight at 4°C. After several washes in buffer, cultures were re-sliced into 30 μm sections. Free-floating sections were washed several times in either 0.1 M PBS or in 50 mM tris-buffered saline (TBS) containing 0.1% Triton X-100, incubated in a blocking buffer [0.5% Triton X-100, 5% bovine serum albumin (BSA) in 50 mM TBS] for 30 min at RT, and subsequently incubated with the primary antibody (diluted in 0.1% Triton X-100, 1% BSA in 0.05 M TBS] for 2 days at RT. Rabbit anti-calretinin (1:500, Swant Cat# CR 7697, RRID: AB_2619710) was used as the primary antibody. After several washing steps, sections were incubated with the appropriate secondary Alexa-conjugated antibody (1:2,000, Invitrogen) for 4 h at RT, counterstained with Hoechst (1:5,000, Sigma–Aldrich) to visualize nuclei, and mounted in Fluorescence Mounting Medium (Dako, Agilent Technologies).

### Quantification of the Sprouting Response

To quantify the expansion of the associational or commissural fiber plexus from the inner into the denervated outer molecular layer, confocal image stacks (EZ-C1, Nikon) of middle sections from re-sliced tissue cultures were captured (10× objective; pinhole size: 30 μm). Using the counterstains to visualize the cytoarchitecture of the DG, the width of the total molecular layer was determined from the outer border of the granule cell layer to the hippocampal fissure using the line tool in ImageJ (Schneider et al., 2012). This was done at three locations (suprapyramidal blade, crest, and infrapyramidal blade of the granule cell layer) and for each dentate gyrus separately. Using an intensity plot profile of calretinin immunofluorescence labeling along the drawn lines, the percentage of the molecular layer covered by the calretinin-positive fiber plexus was calculated for the three locations in control and denervated cultures and subsequently averaged per dentate gyrus. The width of the calretinin plexus in the DG of young as well as old non-denervated cultures was set to one and compared to the width of the calretinin plexus in the DG of young as well as old denervated cultures. This algorithm ensured sampling of the supra- and infrapyramidal blades of the DG as well as the crest region and, thus, yielded an overall parameter of the extent of sprouting.

### Statistics

Statistical analysis was done using GraphPad Prism 6 Software. The Mann–Whitney test was used and significance levels were set at: *p < 0.05; ** *p* < 0.01, ***p < 0.001.

## Results

### Entorhinal Cortex Lesion (ECL) *In vitro* Leaves the Dentate Gyrus Intact and Denervated

**Figure 1:**
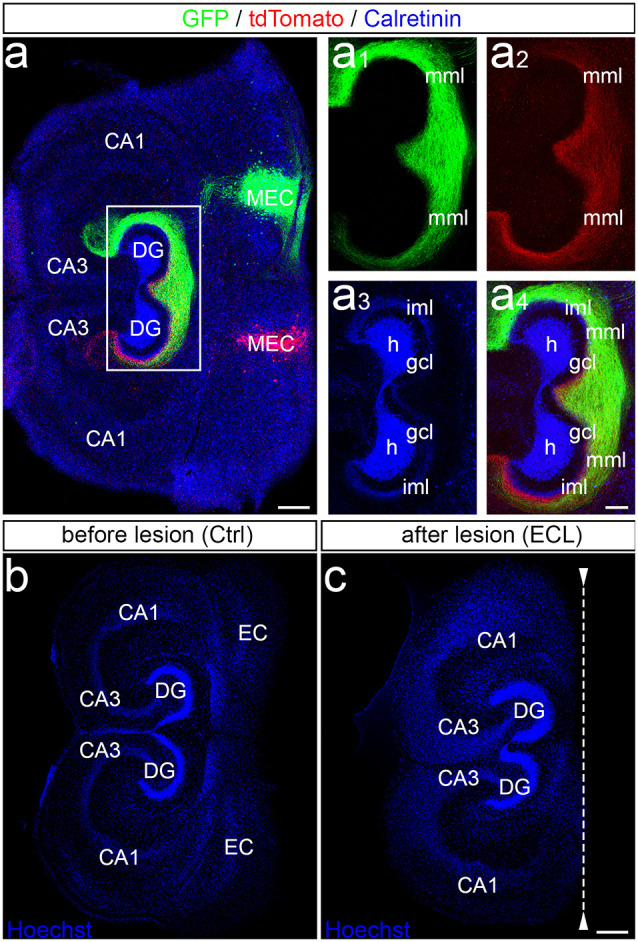
Entorhinal projection and entorhinal lesion *in vitro*. **(A)** Viral tracing was employed to visualize the entorhino-hippocampal projection in double cultures. One medial entorhinal cortex (MEC) was injected with an adeno-associated virus transducing a green construct (AAV2-hSyn-GFP) and the other MEC was injected with a virus transducing a red construct (AAV2-hSyn-tdTomato). In addition, calretinin-immunofluorescence labeling was used to visualize the mossy cells and their axons (blue; overexposed channel to visualize the axons in the inner molecular layer). CA3, CA1, hippocampal subfields CA3, CA1; DG, dentate gyrus; h, hilus; gcl, granule cell layer; iml, inner molecular layer; mml, middle molecular layer. Scale bar: 200 μm. **(A1–A4)** Higher magnification of the dentate gyrus (DG) showing the termination pattern of green MEC axons **(A1)**, red MEC axons **(A2)**, calretinin-immunolabeled axons **(A3)**, and of all three projections **(A4)**. Scale bar: 100 μm **(A1–A4)**. **(B,C)** Entorhinal denervation was performed under visual control. A section (30 μm) of an old (DIV 28) double entorhino-hippocampal tissue culture is illustrated before **(B)** and after **(C)** lesion. Sections were fixed and nuclear staining with Hoechst was used to show that the entorhinal lesion leaves the DG and hippocampus proper intact. EC, entorhinal cortex. Scale bars: 250 μm **(B,C)**.

Entorhino-hippocampal double cultures were generated with the two hippocampi arranged in parallel (Figure 1). Following injection of AAV-viruses into the entorhinal cortex, the bilateral entorhino-hippocampal projection can be visualized (Figure 1A). Note that entorhinal axons from both sides readily intermingle in the “entorhinal zone” of the dentate gyrus (Figures 1A1–A4). Lesions of such cultures are performed under visual control and the EC is cut away, leaving the DG and the hippocampus proper intact (Figures 1B,C). To ensure complete separation from the EC, the EC is removed from the culture dish. Time-lapse imaging of such virally-traced single and double cultures revealed that this procedure reliably and completely removes entorhinal axons from the dentate gyrus (Del Turco et al., 2019; Hildebrandt-Einfeldt et al., 2021).

### Calretinin-Immunolabeling Reveals a Robust Commissural-Associational Sprouting Response in Young Cultures

We previously demonstrated that calretinin-immunofluorescence can be used to quantify collateral sprouting of commissural-associational mossy cell axons after entorhinal denervation (Del Turco et al., 2019). We have now used this approach again to quantify and compare the sprouting of commissural-associational mossy cell axons in young and old cultures in order to find out whether or not the sprouting response *in vitro* is as maturation-dependent, as it is *in vivo* (Gall and Lynch, 1980).

**Figure 2:**
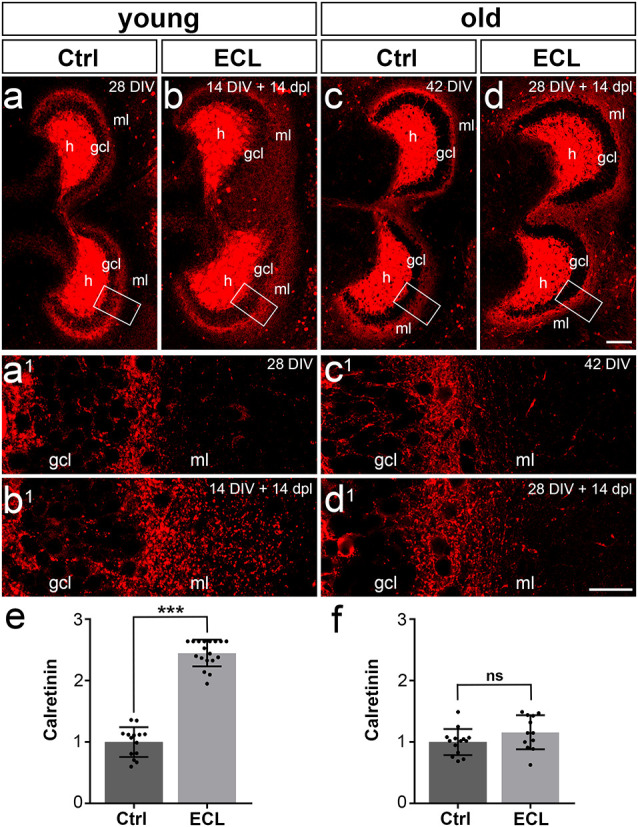
Commissural/associational sprouting is attenuated in cultures lesioned at 28 days *in vitro*. **(A–D)** Double cultures of wild type mouse hippocampus immunolabeled for calretinin (red). **(A)** Overview of a non-denervated young double culture at DIV 14 (control, Ctrl). Calretinin-positive mossy cells were found in the hilus (h) of the culture and mossy cell axons were present in the inner part of the molecular layer (ml). **(B)** Fourteen days post-lesion (dpl) mossy cell axons were found throughout the ml. **(A1,B1)** Higher magnification of the boxed areas in **(A,B)** reveals a robust ingrowth of mossy cell axons into the outer part of the ml (**A1** compared to **B1**) after entorhinal cortex lesion (ECL). **(C)** Overview of a non-denervated old double culture at DIV 28 (Ctrl). Calretinin-positive mossy cells were found in the hilus (h) of the culture, while mossy cell axons were found in the inner part of the molecular layer (ml). **(D)** Fourteen days post-lesion (dpl) mossy cell axons in old cultures stayed within the inner zone of the molecular layer. **(C1,D1)** Higher magnification of the boxed areas in **(C,D)** reveals only a very limited sprouting response (**C1** compared to **D1**) after ECL. Compared to young cultures (**B1** compared to **D1**), the sprouting response is greatly attenuated. **(E)** Quantitative analysis of the axonal sprouting response into the ml in young cultures. Control (Ctrl) was set to 1. A highly significant sprouting response was seen in denervated young cultures compared to non-denervated control cultures (Ctrl: 1.00 ± 0.24, ECL: 2.45 ± 0.22). **(F)** Quantitative analysis of the axonal sprouting response into the ml in old cultures. Control (Ctrl) was set to 1. No significant (ns) sprouting response was seen in denervated old cultures compared to non-denervated control cultures (Ctrl: 1.00 ± 0.21, ECL: 1.16 ± 0.28). Mann–Whitney test; mean ± SD; ***p < 0.001; *n* = 14–18 hippocampi per group in young cultures (data of young cultures from Del Turco et al., 2019) and *n* = 12–14 hippocampi per group in old cultures; gcl, granule cell layer. Scale bars: 100 μm **(A–D)**; 25 μm **(A1–D1)**.

The set of “young” cultures was kept *in vitro* for 14 days before EC-lesion (Del Turco et al., 2019). After a period of 14 days during which sprouting occurred, cultures were immunolabeled (Figures 2A,B) and the expansion of the calretinin-positive axon plexus into the denervated zone was determined. In line with our previous work (Prang et al., 2003; Del Turco and Deller, 2007; Del Turco et al., 2019), a robust ingrowth of calretinin-positive axons into the denervated entorhinal zone was seen bilaterally (Figures 2A–A1,B–B1). Sprouting calretinin-positive axons often reached the hippocampal fissure. The width of the expanded fiber plexus was ~245% of the width of controls (Figure 2E).

### Calretinin-Immunolabeling Reveals an Attenuated Commissural-Associational Sprouting Response in Older Cultures

Next, we analyzed cultures that had been allowed to mature for 28 days *in vitro* before EC-lesion. After another 14 days *in vitro* during which collateral sprouting was allowed to occur, cultures were immunolabeled and the expansion of the calretinin plexus into the denervated zone was analyzed (Figures 2C,D). In contrast to the strong and robust sprouting response we regularly observed in 14 DIV cultures (Figures 2A,B), a considerably attenuated response was observed. In the older cultures, the majority of calretinin-positive axons stayed in the inner molecular layer (Figures 2C1,D1) and did not enter the denervated zone. Although a slight trend towards an enlarged calretinin plexus was present, this expansion into the denervated zone was not significant (Figure 2F). Thus, aged cultures did not show the robust sprouting response characteristic of young cultures.

### Adeno-Associated Viral Tracing in Combination With Time-Lapse Imaging of Sprouting Commissural Fibers Confirms the Attenuated Sprouting Response in Older Cultures

**Figure 3:**
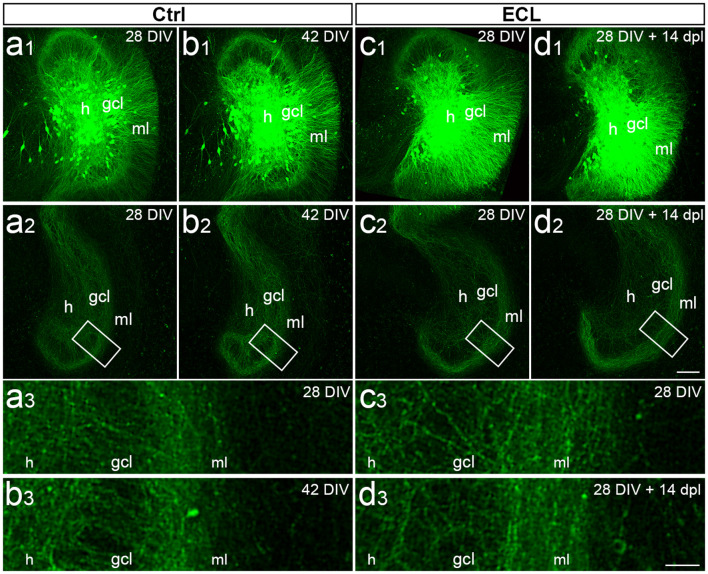
Viral tracing and time-lapse imaging confirm attenuated sprouting response of older cultures. **(A–D)** Imaging of old (DIV 28) entorhino-hippocampal double cultures without (Ctrl; **A,B**) and with entorhinal cortex lesion (ECL; **C,D**). At DIV 4 both sets of cultures received an injection of AAV2-hSyn1-GFP into one hilus (h). On 28 days *in vitro* (DIV) a subset of cultures served as non-lesioned time-matched controls **(A,B)**, whereas a second subset of cultures received an ECL **(C,D)**. **(A1,B1)** Images of the same virus injected control culture at DIV 28 **(A1)** and DIV 42 **(B1)**. Commissural axons in the contralateral dentate gyrus are illustrated at DIV 28 **(A2)** and DIV 42 **(B2)** respectively. The boxed areas are shown at higher magnification **(A3,B3)**, illustrating minimal changes occurring during the 14 days observation period. **(C1,D1)** Images of the same virus injected culture at DIV 28 **(C1)** and 14 days post-lesion (dpl) **(D1)**. Commissural axons in the contralateral dentate gyrus are shown at DIV 28 **(C2)** and DIV 28 + 14 dpl **(D2)**. The boxed areas are shown at higher magnification **(C3,D3)** illustrating no major differences between DIV 28, DIV 42, and 14 dpl. gcl, granule cell layer. Scale bar: 100 μm (**A1–D1** and **A2–D2**); 25 μm **(A3–D3)**.

To qualitatively confirm the data obtained with calretinin-immunolabeling in older cultures, a second approach was used. Adeno-associated viral tracing was employed to visualize the commissural projection using an AAV2-hSyn1-GFP. This virus was microinjected into the hilus and commissural axons were visualized at day 28 *in vitro*. Cultures were divided into two sets, i.e., control (Figures 3A1–A2,B1–B2) and lesioned (Figures 3C1–C2,D1–D2) cultures. Both sets were imaged on day 28 and a second time 14 days later. In the control cultures, the commissural fiber plexus was comparable at both time points (Figures 3A2,B2) and, as expected, no invasion of these fibers into the denervated zone could be seen (Figures 3A3,B3). In the lesioned cultures, a similarly robust commissural plexus was present prior to denervation (Figures 3C2,D3) and—similar to what we had observed in the calretinin-immunolabeled cultures—after 14 days post-lesion no or only minimal sprouting of commissural axons into the denervated zone could be seen (Figures 3C3,D3). Thus, viral tracing in combination with time-lapse imaging confirmed that older cultures do not exhibit a robust sprouting response of commissural axons into the denervated out molecular layer.

## Discussion

Lesion-induced functional and structural reorganization of the CNS is arguably one of the most important naturally occurring repair mechanisms of the brain (Steward, 1991). It comes into play after some kind of structural damage causes the deafferentation of a connected brain region and is independent of the underlying disease or cause (Steward, 1991; Deller and Frotscher, 1997). To study this form of pathology-related brain reorganization the unilateral entorhinal cortex lesion model has been used for several decades (Cotman and Nadler, 1978; Gall and Lynch, 1980; Steward, 1991; Deller, 1997; Deller and Frotscher, 1997). We transferred the classical *in vivo* denervation model to the culture dish using organotypic entorhino-hippocampal tissue cultures (Prang et al., 2003; Del Turco and Deller, 2007). Using this *in vitro* system, we demonstrated early functional adaptations (Vlachos et al., 2012b; Vlachos et al., 2013b), the reorganization of dendritic spines (Vlachos et al., 2012a; Yap et al., 2020), and the remodeling of dendrites (Willems et al., 2016) as well as collateral sprouting of surviving afferents (Prang et al., 2003; Del Turco et al., 2019). All of these denervation-induced adaptations *in vitro* were homeostatic in nature aiming for a recovery of function.

Of note, the ability of the brain to reorganize itself and to remodel its connections is age or rather maturation-dependent. This has been shown very early for the *in vivo* entorhinal denervation model in a series of articles by Gall, Lynch, and co-workers (Gall and Lynch, 1980, 1981; Gall et al., 1980; McWilliams and Lynch, 1983). These authors showed that young rats show a much stronger sprouting response compared to older rats. Specifically, in young rats commissural/associational fibers sprout from the inner molecular layer all the way to the hippocampal fissure. With maturation, however, sprouting becomes less intense and in adult rats, the expansion of the commissural/associational axon plexus is very limited (Deller et al., 1996b; Frotscher et al., 1997). The situation is similar in adult mice *in vivo*, which show only very limited sprouting of commissural/associational axons from the inner molecular layer into the denervated entorhinal zone (Del Turco et al., 2003; Deller et al., 2007). Thus, whereas in the young rat sprouting of commissural/associational axons is extensive, in the adult rat as well as in the adult mouse sprouting of commissural/associational axons is limited and the sprouting response appears to respect the laminar boundaries of the dentate gyrus (Frotscher et al., 1997).

**Figure 4:**
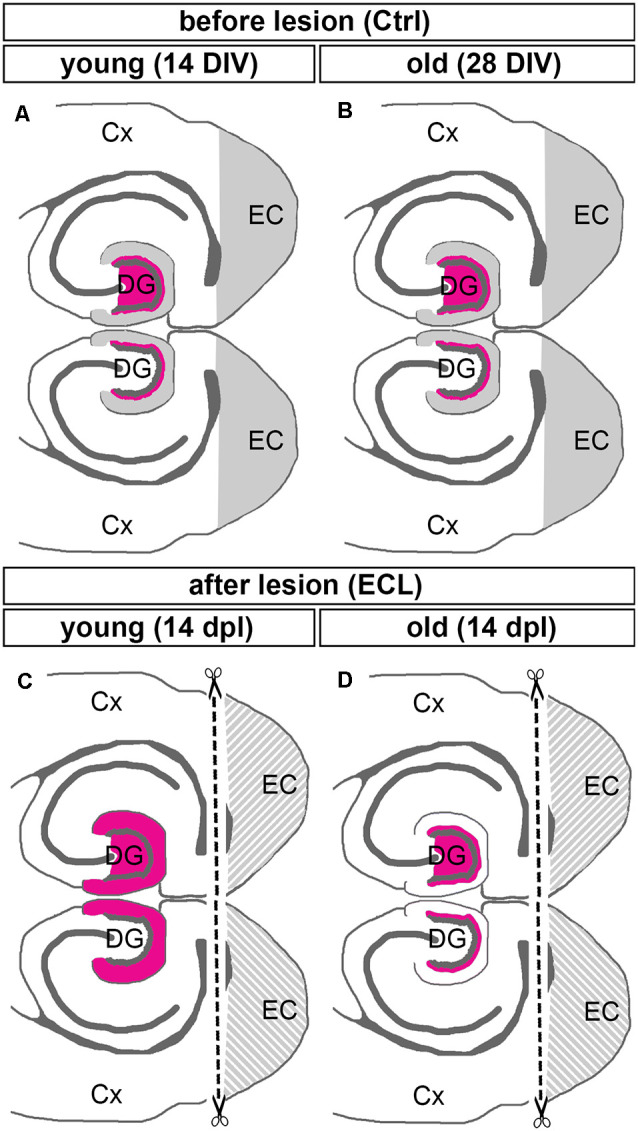
Summary diagram illustrating maturation-dependent differences in the sprouting of commissural/associational mossy cell axons after entorhinal lesion. **(A,B)** Before lesion (control, Ctrl) DIV 14 (young) and DIV 28 (old) double entorhino-hippocampal tissue cultures showed an organotypic and layer-specific entorhinal innervation of the outer molecular layer of the dentate gyrus (DG, gray). Commissural/associational axons arising from hilar mossy cells terminate in the inner molecular layer of the dentate gyrus (magenta; only the mossy cell projection of the upper DG is shown). **(C,D)** Fourteen days post-lesion (dpl) a robust sprouting response is seen in young cultures **(C)**. In these cultures, commissural/associational axons grow into the denervated zone up to the hippocampal fissure. This corresponds to the juvenile sprouting response reported for commissural/associational axons *in vivo*. **(D)** In contrast, in older cultures commissural/associational axons stayed largely in their home territory and did not invade the denervated zone. This corresponds to the adult pattern of sprouting of commissural/associational axons seen *in vivo*. Note, only the mossy cell projection of the upper DG is shown in **(C,D)**. Cx, cortex; EC, entorhinal cortex.

We now wondered whether a similar maturation-dependent phenomenon could be observed *in vitro*. This appeared to be likely since organotypic slice cultures continue to mature *in vitro* (Woodhams et al., 1993; Frotscher et al., 1995; Li et al., 1995; Dailey and Smith, 1996; Ziv and Smith, 1996; Prang et al., 2001) and the ability of axons to regenerate following axotomy is lost after ~10 days *in vitro* (Li et al., 1995; Prang et al., 2001; Del Turco and Deller, 2007). We, therefore, speculated that young cultures should show a strong sprouting pattern characteristic of an immature brain, whereas older cultures that have reached a *bona fide* “adult stage” should also show an adult sprouting pattern, i.e., only limited sprouting. Indeed, our study demonstrates that this aspect of the *in vivo* model is also present in the culture dish: “young” lesioned organotypic slice cultures show a robust and rapid sprouting response, similar to immature rats *in vivo*, whereas “older” lesioned organotypic slice cultures show a very restricted sprouting response, similar to the one seen in mature rats or mice (Figure 4).

What could be the underlying mechanisms of these two different sprouting patterns in young and old cultures? Both cell-intrinsic as well as extrinsic factors could play a role and could restrict sprouting in older cultures. In the first case, maturation-dependent changes in the expression of growth-associated proteins, such as GAP-43, MARCKS, or CAP-23 could play a role, since these intrinsic determinants of axonal growth of neurons are downregulated in a cell-autonomous manner with maturation (Caroni, 1997). Thus, changes in the propensity of a neuron to grow could depend on levels of growth-associated proteins in the sprouting neurons, e.g., mossy cells. Overexpressing these proteins in aged cultures will show whether this plays an important role in limiting the sprouting response. Alternatively, the maturation of the substrate into which the axons grow could play a role (Deller et al., 1997, 2000; Haas et al., 1999; Thon et al., 2000). The extracellular matrix also changes with maturation and the balance of growth–promoting and growth–inhibiting molecules inside the extracellular meshwork may change (Margolis et al., 1996; Schwartz and Domowicz, 2018). Indeed, we have previously shown that a number of growth–inhibitory extracellular matrix proteins are expressed in the mature extracellular matrix following denervation, which could limit the growth response of commissural/associational axons in adult rats and mice (Deller et al., 1997, 2000; Haas et al., 1999; Thon et al., 2000). It is a plausible explanation that maturation-dependent changes in the growth substrate play an important role and prevent commissural/associational axons from growing into the denervated area. Finally, a combination of these two and other factors is also conceivable.

The clinical consequences of maturation-dependent differences in the sprouting response have been discussed in the literature: young children who have received a limited traumatic brain injury or who had to undergo localized brain surgery during their early life show a faster and more extensive brain reorganization compared to adults (Schneider, 1979; Steward, 1991). However, as pointed out, the stronger sprouting response of the young brain may also have some significant downsides, since aberrant connections may also form leading to e.g., involuntary movements (Schneider, 1979). As seen in the *in vitro* experiments with young cultures (Prang et al., 2003; Del Turco et al., 2019) and also in young rats *in vivo* (Gall and Lynch, 1980), an abnormal connectivity is indeed established in the young denervated dentate gyrus: commissural/associational axons, which are normally restricted to the inner molecular layer of the dentate gyrus grow out and cover the entire molecular layer of the dentate gyrus, including the outer layers normally reserved for entorhinal axons. Although this kind of sprouting is likely to provide some benefit to the denervated granule cells (Steward, 1991), information processing from the entorhinal cortex to the hippocampus is not re-established. In contrast, in the adult mouse and rat commissural/associational sprouting is limited (Frotscher et al., 1997) and—at least in the rat—sprouting of homologous crossed entorhinal axons occurs (Steward, 1976; Deller et al., 1996a). Although weaker in strength, this mature form of sprouting appears to be more specific than the sprouting seen in the young.

In sum, sprouting is an important naturally occurring repair mechanism of the brain (Steward, 1991) that has been exploited for rehabilitation purposes (Fawcett, 2009; Garcia-Alias et al., 2009; Maier et al., 2009; Filli and Schwab, 2015). Although at first glance collateral sprouting appears to be the same phenomenon in the injured young and the injured adult brain, maturation-dependent differences in growth propensity, e.g., growth-associated proteins, as well as differences in guidance cues, e.g., extracellular matrix molecules, may cause considerable differences in the extent of the sprouting response seen at different ages. The *in vitro* entorhinal denervation model reflects the maturation-dependent changes in sprouting observed in experimental animals * in vivo* as well as in human patients and offers the chance to study and understand such maturation-dependent differences better in the future.

## Data Availability Statement

The original contributions presented in the study are included in the article, further inquiries can be directed to the corresponding author.

## Ethics Statement

The generation of organotypic entorhino-hippocampal tissue cultures from C57Bl6/J mice was performed in accordance with the German animal welfare law and had been declared to the Animal Welfare Officer of Goethe-University, Faculty of Medicine (Wa-2014-35). Mice were bred and housed at the animal facility of the Goethe-University Hospital Frankfurt, and were maintained on a 12 h light/dark cycle with food and water available *ad libitum*. Every effort was made to minimize distress and pain of animals.

## Author Contributions

MP, VB, and LH-E acquired and analyzed the data. DD and TD conceived and supervised the study. DD, MP, and TD wrote the manuscript. All authors were involved in data interpretation and critically revising the manuscript. All authors read and approved the final manuscript. All authors contributed to the article and approved the submitted version.

## Conflict of Interest

The authors declare that the research was conducted in the absence of any commercial or financial relationships that could be construed as a potential conflict of interest.
